# Discontinuities in understanding follicular development, the ovulatory cycle and the oviposition cycles in the hen: Advances, opportunities, slow downs and complete stops

**DOI:** 10.3389/fphys.2022.1023528

**Published:** 2022-10-03

**Authors:** Colin G. Scanes

**Affiliations:** Department of Biological Science, University of Wisconsin Milwaukee, Milwaukee, WI, United States

**Keywords:** ovulation cycle, oviposition, follicular development, ahemoral, hen reproduction

## Abstract

There has been considerable progress in understanding follicular development, the ovulatory cycle and the oviposition cycles in the hen. In particular, there have been tremendous advances in understanding follicular development and recruitment of follicles to the hierarchy of large yellow follicles. There is a need to continue to follow the earlier literature while employing present techniques. Early work allowed an understanding of the ovulation and oviposition cycles. Models for ovulation were developed. However, while these have no passed the test of time, there is no present model that fully accounts to the cycles. Earlier work employed ahemoral light cycles to examine ovulation and oviposition cycles. Recent work has demonstrated that clock genes are expressed in the ovary. The control of incubation by prolactin has been largely elucidated in turkeys. There is evidence that other endocrine glands influence female reproduction in birds including the adrenal cortex, thyroid and pineal. However, there is much that remains to be fully understood.

## Introduction

My interest in the physiology of reproduction in birds dawned with my being a member of the team that developed a radioimmunoassay for chicken luteinizing hormone (LH) (Follett et al., 1972); this being the first assay for a non-mammalian hormone. This and other such assays were used, for instance, to determine changes in plasma concentrations of LH along with progesterone during the ovulatory cycle of chickens (Furr et al., 1973) and the circadian basis of photoperiodic induction of LH release in a wild bird (Follett et al., 1974). Brian Follett went on to an exemplary research career deducing much of the mechanism of photoperiodism in birds.

It is appropriate, 50 years later, to consider what has been learned on the ovulatory or oviposition cycles of chickens and turkeys and what questions remain needing to be addressed. Among the unique features of female reproduction in poultry and other birds are the following:

• A hierarchy of yellow (yolk filled) follicles with a new follicle recruited on a daily basis.
• Ovulation of clutches of eggs with the time of ovulation occurring later in the day as the sequence progresses
• The ovum passing through the oviduct where it acquires albumen (egg white), membranes and a calcareous shell. Once this is complete, the egg is released from the oviduct in the process of oviposition (egg laying). This will not be covered except where is impacts ovulation, oviposition and their timing.
• Broodiness and the incubation of eggs.

## Development of follicles

There is a hierarchy of yellow (yolk filled) follicles with follicles increasing in size to a maximum diameter of 2.5 cm due to their filling with yolk. There is also maturation of the granular and thecal cells. The largest follicle will be the first to be ovulated and then the next largest. A new follicle recruited on a daily basis.

Alan Johnson’s laboratory have performed a series of studies on the recruitment and development of the ovarian follicles. Not only does this provide a comprehensive account of follicular development but also the studies themselves were exquisite. The recruitment of small (pre-hierarchal) follicles involves follicle stimulating hormone (FSH) and other factors such as growth factors increasing FSH receptors by granulosa cells from pre-hierarchal follicles. In turn, there are increases in the following:

1) Formation of cyclic adenosine monophosphate (cAMP).
2) Expression of steroidogenic acute regulatory protein (STAR).
3) Production of progesterone by granulosa cells (Kim and Johnson, 2018).

Effects of growth factors include the following:

**Bone morphogenetic protein 4 (BMP4)** increased expression of the FSH receptor in undifferentiated granulosa cells from pre-hierarchal follicles (Kim et al., 2013). Similarly, (BMP6 increased responsiveness to FSH by granulosa cells from pre-hierarchal follicles (Ocón-Grove et al., 2012). Conversely, BMP2 prevented FSH receptor expression by either transforming growth factor β (TGF β) or FSH by undifferentiated granulosa cells from pre-hierarchal follicles (Haugen and Johnson, 2010).
**Transforming growth factor β1 (TGFβ1)** increased expression of vascular endothelial growth factor A (VEGF) and its receptor, VEGF receptors (VEGFR) in granulosa cells from prehierarchal follicles (Kim et al., 2016). In turn, VEGF and VEGFR induce angiogenesis and consequently facilitate follicular growth and the deposition of yolk precursors.
**BMP6** also increased the expression of anti-Müllerian hormone (AMH) by granulosa cells from pre-hierarchal follicles (Ocón-Grove et al., 2012). In the presence of FSH, BMP4 increased AMH expression by undifferentiated granulosa cells from pre-hierarchal follicle (Kim et al., 2013). Moreover, the effect of BMP4 was blocked in the presence of TGFα or noggin (Kim et al., 2013).

## Ovulation and oviposition cycles

For every ovum to be ovulated, there is a surge in circulating concentrations of LH and progesterone. A very few pre-ovulatory surges in circulating concentrations of LH and progesterone were not associated with an egg laid in turkeys (Liu et al., 2001a; Liu et al., 2001b). The interval between LH/progesterone surges is increased late in reproductive period (Liu et al., 2002). A positive feedback loop exists with LH stimulating production of progesterone by granulosa cells particularly those in the largest follicle and progesterone increasing LH release.

Progesterone induces the pre-ovulatory LH surge (Rothchild and Fraps, 1949; Wilson and Sharp, 1975). The effect of progesterone requires the presence of estrogen. Progesterone only provokes a LH surge when ovariectomized hens received estradiol administration of daily prior to challenge (Wilson and Sharp, 1976).

## Timing of oviposition and ovulation

The interval between ovipositions is 24–27 h [chickens: (Attwood, 1929) also see Table 1; turkeys: 26.8 h: (Liu et al., 2001a); 26 h: (Brady et al., 2019)]. The interval varies with the length of a sequence in the laying hen (Attwood, 1929) with greater intervals with the shorter sequences (Table 1). Assuming that the interval between oviposition and ovulation of the next ovum in the sequence remains constant at about 30 min (Warren and Scott, 1935), the duration the ovum spends in the oviduct is markedly less in long sequences (Table 1) tending to less 24 h.

**TABLE 1:** Effect of sequence length on interval between ovipositions [from or calculated from Attwood (1929)].

Sequence length (number of eggs in a sequence)	Interval between ovipositions (h)	Duration in oviduct^a^ (h)
2	28.0	26.4
3	26.8	27.5
4	25.9	25.4
5	25.6	25.1
8	24.6	24.1
11	24.7	24.2

^a^Calculated assuming interval between oviposition and ovulation of 30 min (Warren and Scott, 1935).

**FIGURE 1:**
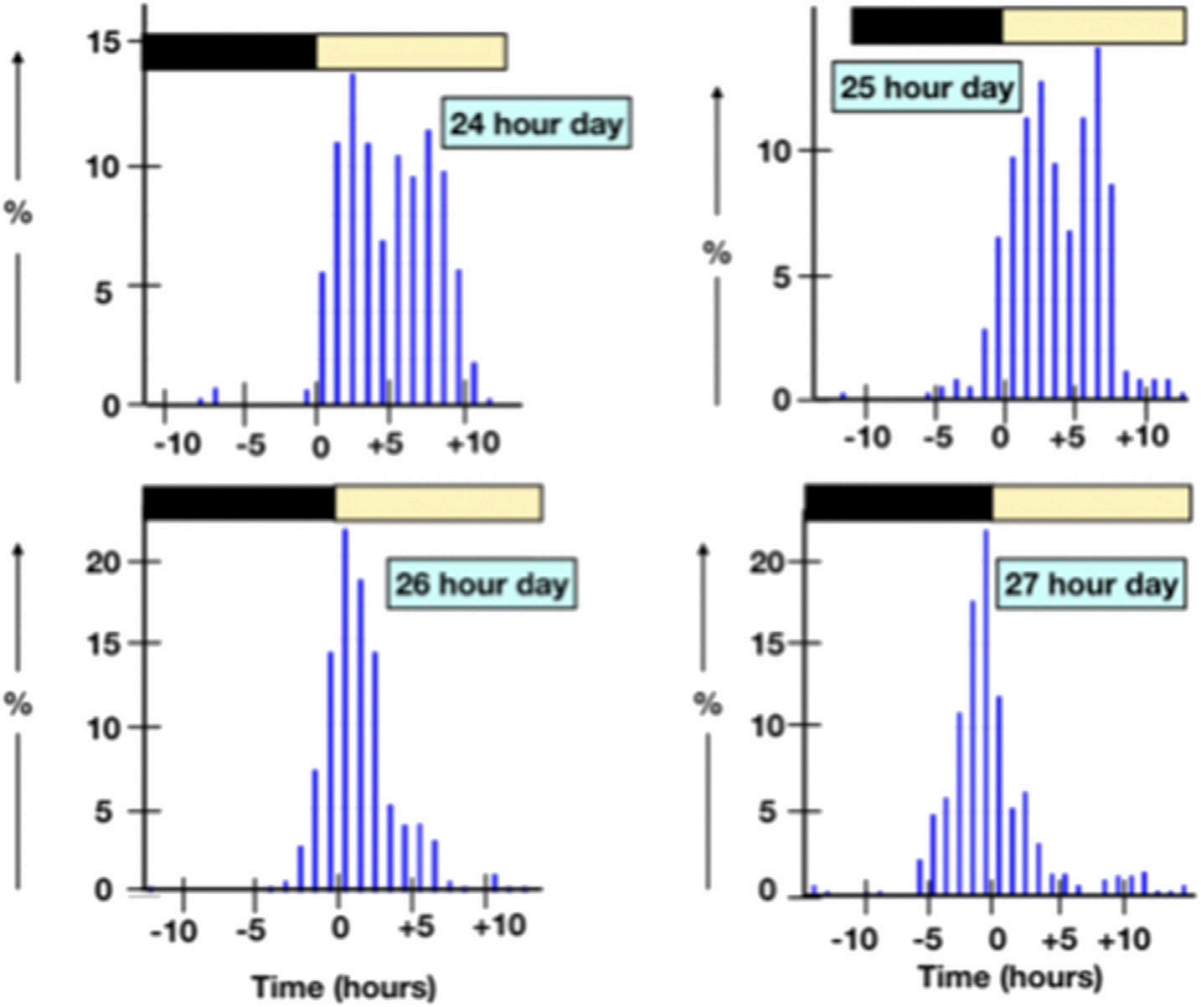
Shifts in the time of oviposition with ahemoral light cycles.

The interval between oviposition and the next oviposition reflects the time an ovum spends in the oviduct together with the approximately the time between oviposition and the next ovulation (Table 1) [calculated from data in Biellier and Ostmann (1960)]. The interval between ovipositions is greater in short sequences (Table 2) and is longer with long ahemoral light cycles (Table 1) (Morris, 1973; other data calculated from data in Biellier and Ostmann, 1960). With ahemoral light cycles of progressively greater than 24 h, the timing of oviposition is earlier; migrating from during the photophase to the end of the scotophase (calculated from data in Biellier and Ostmann, 1960) (Figure 1; Tables 2, 3). It is unclear how the light/dark cycle influences the duration that an ovum spends in the oviduct?

**TABLE 2:** Effect of ahemeral light/dark cycles on interval between ovipositions (based on or calculated from data in Biellier and Ostmann, 1960; Morris, 1973).

Light (photophase)/dark (scotophase) cycles	Length of “day”^a^ (h)	Interval between ovipositions (h)	Duration of ovum in the oviduct^b^ (h)
Biellier and Ostmann (1960)			
10.5L:10.5D	21	26.4	25.9
11L:11D	22	27.5	27.0
11.5L:11.5D	23	26.1	25.6
12L:12D	24	26.4	25.9
19L:19D	38	32.3	31.8
21L:21D	42	34.9	34.4
Morris (1973)			
14L:10D	24	24.9	24.4
14L:13D	27	27.1	26.6
14L:16D	30	29.0	28.5

^a^Photophase plus scotophase.

b Calculated assuming interval between oviposition and ovulation of 30 min (Warren and Scott, 1935).

**TABLE 3:** Effect of ahemeral light/dark cycles on the timing of ovipositions (calculated from data in Biellier and Ostmann, 1960.

Light (photophase)/dark (scotophase) cycles	% Ovipositions during photophase	Earliest time for oviposition^a^ ^,^ ^b^	Time for latest of ovipositions^a^ ^,^ ^c^	Mean time of oviposition^a^ ^,^ ^b^
10.5L:10.5D	67.3	+5 to +6	−8 to −7	+7.9
11.L:11.D	86.8	+4 to +5	−9 to −8	+8.3
11.5L:11.5D	95.5	+2 to +3	+10 to +11	+4.8
12L:12D	98.4	0 to +1	+9 to +10	+4.9
12.5L:12.5D	88.1	−1 to 0	+7 to +8	+4.6
13L:13D	74.1	−2 to −3	+3 to +4	+2.2
13.5L:13.5D	35.9	−3 to −4	+3 to +4	−0.7

^a^Time for earliest ovipositions (>5% of total).

^b^Time in hours from onset of the photophase.

^c^Time for latest ovipositions (<5% of total).

In laying hens, ovulation occurs about 30 min following oviposition of the previously ovulated ovum (Warren and Scott, 1935; Melek et al., 1973). There is some difference between the interval oviposition and ovulation irrespective of the photoperiod; this being 24 min for laying hens on a 14L:10D photoperiod and 36 min for hens on ahemeral light schedule (14L:13D) (Melek et al., 1973). What is not clear is the mechanisms for the cross talk between the ovary and oviduct?

The corollary to ovulation occurring 30 min after oviposition of the previous ovum in a sequence is that the surge in circulating concentrations of LH and progesterone occurs about 6 h before oviposition of the previous ovum. What is not clear is how the ovum/pituitary anticipate oviposition of the previous ovum in the sequence? What is the cross talk between the ovary and/or its hypothalamic pituitary control (ovulation) and the oviduct (oviposition of the previously ovulated ovum)?

Laying hens have been selected for reduced intervals between ovipositions under continuous lighting (24L:0D) (Gow et al., 1985). These hens also exhibited reduced intervals between surges in circulating concentrations of LH (Gow et al., 1985). The timing of the surge in circulating concentrations of LH and progesterone occurs at a specific time of day. For instance, in the domestic duck, the first pre-ovulatory surge in circulating concentrations of LH and progesterone in a sequence occurs at the beginning of the scotophase on a 16L:8D photoperiod and 2 h into the scotophase on a 11L:13D photoperiod (Wilson et al., 1982). The first LH surge of a sequence occurs at the beginning of the scotophase in hens on a 14L:10D photoperiod (Johnson and van Tienhoven, 1984). The pre-ovulatory LH surge in chickens occurs at the beginning of the scotophase (Wilson et al., 1985). If the timing of the scotophase is advanced, there is some increase in circulating concentrations of LH, albeit not a full LH surges (Wilson et al., 1985).

## Model for the ovulatory cycle

Both oviposition and ovulation occur only during a period of 8–10 h during the day in chickens and turkeys (on a photoperiod of 14L:10D) with the first ovulation in a sequence occurring 8–9 h after the subjective dusk and the last ovulation of a sequence occurring 18–19 h after the subjective dusk. This was called the open-period (Fraps, 1954; Fraps, 1965). The corollary is that the LH/progesterone surge for the first ovulation in a sequence occurs about 3 h into the scotophase. As the sequence progresses, the surge occurs later and later until the last surge occurs 12 h from the subjective dusk. A further corollary is that the LH/progesterone surge is limited to this open period. What was not clear was what was the open period? 1) Was it the ability of the ovary to produce progesterone? There is not evidence for this. 2) Was it the ability of the hypothalamus/pituitary gland to respond to progesterone positive feedback? There is no evidence for this either. Occam’s razor Was it some other mechanism?

An alternate model for the sequence of ovulations postulated two asynchronous cycles with ovulation only occurring when these were synchronized (Bastian and Zarrow, 1955). What was not clear is what was the physiological bases of each cycle? This would seem to be a non-testable hypothesis as the cycles are not defined.

## Circadian genes

There are multiple circadian genes. For instance, BMAL1 is heterodimeric transcriptional protein and one of the master genes of the circadian clock (Menet et al., 2014). There is expression of Bmal1 together with other circadian genes in the ovary of the laying hen: cryptochrome circadian regulator (Cry1), Clock and period circadian regulator (Per 2). Moreover, there is evidence that the pre-ovulatory LH surge influences expression of circadian genes (Tischkau et al., 2011; Li et al., 2014). Expression of BMAL1 by chicken follicular granulosa cells has been reported with expression increased by vasoactive intestinal peptide (VIP) (Kim and Johnson, 2016). What are not clear include the following: 1) Are circadian genes expressed in the oviduct? 2) How are circadian genes in the ovary and, potentially also, the oviduct entrained following a shift in photoperiod and under ahemoral cycles? Are they entrained by the light dark cycle or the stage of the sequence and, if so, what is the mechanism of entraining expression of the clock genes? Is the expression of the clock genes influenced by phase advancing or phase delaying the LH/progesterone surge or the imposition of ahemeral lighting cycles?

## Neuroendocrine control of female reproduction

A series of papers from J. P. Advis’s laboratory provided evidence for noradrenergic, neuropeptide Y (NPY) and dopaminergic effects on gonadotropin releasing hormone (GnRH) release from the median eminence (Contijoch et al., 1990; Contijoch et al., 1992; Contijoch et al., 1993). Norepinephrine stimulated GnRH release from the median eminence from laying hen (Contijoch et al., 1990). Similarly, neuropeptide Y increased *in vitro* GnRH release from hen median eminence (Contijoch et al., 1993). Basal GnRH release from the median eminence of hens subjected to feed withdrawal was increased but decreased in the presence of dopamine (Contijoch et al., 1992). Unfortunately, this group did not appear to have continued research on the reproductive physiology of hen. There is further evidence for both dopaminergic and adrenergic control of preovulatory LH surge with the surge blocked in the presence of the dopamine agonist (apomorphine) or an α adrenergic antagonist (phenoxybenzamine) (Knight et al., 1982). What is still unclear whether additional neuropeptides are involved in the control of the pre-ovulatory surge?

## Recent progress in dissecting the hypothalami-pituitary—ovarian axis

Gene expression in the hypothalamus, pituitary and ovary was compared between during the pre-ovulatory LH/progesterone in a study of turkey hens in Tom Porters’s laboratory. The changes appear to be not those expected. For instance, there is reduced expression of GnRH in the hypothalamus (Brady et al., 2019). Moreover, there is decreased expression of GnRH receptors and increased expression of GnIH receptors in the pituitary gland (Brady et al., 2019). There was also shifts in expression in the preovulatory surge in the ovary such as decreases in LHR in the granulosa of F1 follicle (Brady et al., 2019). What is not clear is how the role for each component of the hypothalami-pituitary—ovarian axis controlling ovulation?

## Broodiness and the incubation of eggs

Our knowledge of the endocrine control of incubation (sitting on eggs) and brooding (care of chicks/poults). Much of this stems for the work of Mohamed El Halawani. Circulating concentrations of prolactin are markedly increased in turkeys during incubation with a decline when the birds are deprived of access to nests (El Halawani et al., 1980). This increase in prolactin is controlled by hypothalamic peptide, vasoactive intestinal peptide (VIP). Immunization of turkeys against VIP prevents the increase in circulating concentrations of prolactin in turkeys (El Halawani et al., 1996; El Halawani et al., 2000). VIP increases prolactin expression and release in turkeys with the effect blocked by dopamine (Sun and El Halawani, 1995; Al Kahtane et al., 2005). Hypothalamic expression of vasoactive intestinal peptides is high in incubating turkeys (Rozenboim et al., 1993). Similarly, there is increased VIP receptor expression in the anterior pituitary glands in turkeys during incubation (Chaiseha et al., 2004). Administration of a dopamine antagonist prevented brooding behavior in poults (Thayananuphat et al., 2011). Unfortunately, Mohamed El Halawai has now retired and is inactive.

## Other endocrine inputs

### The relationship between adrenal cortical hormones and the ovulation cycle

There is evidence from early studies that adrenal cortical hormones influence can influence ovulation. Ovulation was blocked by administration of the glucocorticoid, dexamethasone, 14 h prior to ovulation (Soliman and Huston, 1974). The effect of dexamethasone was overcome by the administration of adrenocorticotropic hormone (ACTH) (Soliman and Huston, 1974). This is consistent with dexamethasone suppressing adrenocorticotropic hormone (ACTH) release either acting directly at the level of the anterior pituitary gland or indirectly by depressing release of corticotropin releasing hormone and/or the releasing hormones for ACTH, namely arginine vasotocin (AVT) from the hypothalamus. Premature ovulation was induced by the following in order of potency: Deoxycorticosterone, progesterone, and, at very high dose, corticosterone (Etches and Cunningham, 1976). Moreover, each agent induced premature oviposition (Etches and Cunningham, 1976; Wilson and Sharp, 1976). In contrast, ovulation was inhibited by either the synthetic glucocorticoid, dexamethasone (Rzasa et al., 1983) or corticosterone (Williams et al., 1985).

### The relationship between thyroid hormones and reproduction

There is evidence for relationships between thyroid hormones and egg laying in poultry with, for instance, plasma concentrations of triiodothyronine decreased during the LH/progesterone surge (Brady et al., 2021). There are decreases in the plasma concentrations of thyroid hormones, triiodothyronine (T_3_) and thyroxine (T_4_) prior to the onset of ovulation during sexual maturation (Sechman et al., 2000). Moreover, T_3_ has been demonstrated to depress plasma concentrations of both LH and estradiol, to induce follicular atresia and to bind to thyroid hormone receptor in ovarian follicles (Sechman et al., 2009; reviewed: Sechman, 2013). What is not clear is whether the shifts in thyroid hormones can be advanced or delayed experimentally?

### The relationship between gonadotropin inhibitory hormone and reproduction

The RFamide peptide, gonadotropin inhibitory hormone (GnIH) depresses both release of LH and FSH and expression of the common alpha and FSH beta gonadotrophin subunit *in vitro* (Ciccone et al., 2004). There are also direct effects of GnIH on the ovary. There is expression of both GnIH and its receptor (GnIHR) in the chicken ovary (Maddineni et al., 2005). Expression of GnIH declines during sexual maturation (Maddineni et al., 2005).

### The relationship between melatonin and reproduction

Melatonin administration increased ovarian expression of both melatonin receptors type 1A (MTNR1A) and melatonin receptors type 1B (MTNR1B) (Hao et al., 2020). There have been studies on the effects on light spectrum on ovarian expression of MTNR1A, MTNR1B and melatonin receptors type 1C (MTNR1C) (Li et al., 2015). Expression of MTNR1A and MTNR1C was greater in hens on monochromatic red (660 nm) than green (560 nm) and blue (480 nm) light in small yellow follicles, F5 and F2 follicles (Li et al., 2015). However, there were no effects on ovarian expression of MTNR1B (Li et al., 2015). A role for gonadal receptors melatonin receptors in seasonal breeding has been proposed in starlings (McGuire et al., 2011).

There is a positive correlation between plasma concentration of melatonin and both hypothalamic concentrations of GnIH and GnIHR during reproductive development in chickens (Zhang et al., 2017).

## References

[B1] Al Kahtane A. Kannan M. Kang S. W. El Halawani M. E. Al KahtAne A. (2005). Regulation of prolactin gene expression by vasoactive intestinal peptide and dopamine in the Turkey: Role of Ca signalling. J. Neuroendocrinol. 17, 649–655. 10.1111/j.1365-2826.2005.01352.x 16159377

[B2] Attwood H. (1929). A study of the time factor in egg production. Poult. Sci. 8, 137–140.

[B3] Bastian J. W. Zarrow M. X. (1955). A new hypothesis for the asynchronous ovulatory cycle of the domestic hen (*Gallus domesticus*). Poult. Sci. 34, 776–788. 10.3382/ps.0340776

[B4] Biellier H. V. Ostmann O. W. (1960). Effect of varying day-length on time of oviposition in domestic fowl. U. Mo. Agr. Exp. Sta. Res. Bull. 747.

[B5] Brady K. Long J. A. Liu H. C. Porter T. E. (2021). Characterization of hypothalamo-pituitary-thyroid axis gene expression in the hypothalamus, pituitary gland, and ovarian follicles of Turkey hens during the preovulatory surge and in hens with low and high egg production. Poult. Sci. 100, 100928. 10.1016/j.psj.2020.12.026 33588341 PMC7896151

[B6] Brady K. Porter T. E. Liu H.-C. Long J. A. (2019). Characterization of gene expression in the hypothalamo-pituitary-gonadal axis during the preovulatory surge in the Turkey hen. Poult. Sci. 98, 7041–7049. 10.3382/ps/pez437 31399736 PMC6870558

[B7] Chaiseha Y. Youngren O. M. El Halawani M. E. (2004). Expression of vasoactive intestinal peptide receptor messenger RNA in the hypothalamus and pituitary throughout the Turkey reproductive cycle. Biol. Reprod. 70, 593–599. 10.1095/biolreprod.103.022715 14568918

[B8] Ciccone N. A. Dunn I. C. Boswell T. Tsutsui K. Ubuka T. Ukena K. (2004). Gonadotrophin inhibitory hormone depresses gonadotrophin alpha and follicle-stimulating hormone beta subunit expression in the pituitary of the domestic chicken. J. Neuroendocrinol. 16, 999–1006. 10.1111/j.1365-2826.2005.01260.x 15667455

[B9] Contijoch A. M. Gonzalez G. Singh H. N. Malamed S. Troncoso S. Advis J. P. (1992). Dopaminergic regulation of luteinizing hormone-releasing hormone release at the median eminence level: Immunocytochemical and physiological evidence in hens. Neuroendocrinology 55, 290–300. 10.1159/000126128 1354335

[B10] Contijoch A. M. Johnson A. L. Advis J. P. (1990). Norepinephrine-stimulated *in vitro* release of luteinizing hormone-releasing hormone (LHRH) from median eminence tissue is facilitated by inhibition of LHRH-degrading activity in hens. Biol. Reprod. 42, 222–230. 10.1095/biolreprod42.2.222 2186814

[B11] Contijoch A. M. Malamed S. McDonald J. K. Advis J. P. (1993). Neuropeptide Y regulation of LHRH release in the median eminence: Immunocytochemical and physiological evidence in hens. Neuroendocrinology 57, 135–145. 10.1159/000126353 8479609

[B12] El Halawani M. E. Burke W. H. Dennison P. T. (1980). Effect of nest-deprivation on serum prolactin level in nesting female turkeys. Biol. Reprod. 23, 118–123. 10.1095/biolreprod23.1.118 7417659

[B13] El Halawani M. E. Pitts G. R. Sun S. Silsby J. L. Sivanandan V. (1996). Active immunization against vasoactive intestinal peptide prevents photo-induced prolactin secretion in turkeys. Gen. Comp. Endocrinol. 104, 76–83. 10.1006/gcen.1996.0143 8921358

[B14] El-Halawani M. E. Whiting S. E. Silsby J. L. Pitts G. R. Chaiseha Y. (2000). Active immunization with vasoactive intestinal peptide in Turkey hens. Poult. Sci. 79, 349–354. 10.1093/ps/79.3.349 10735201

[B15] Etches R. J. Cunningham F. J. (1976). The effect of pregnenolone, progesterone, deoxycorticosterone or corticosterone on the time of ovulation and oviposition in the hen. Br. Poult. Sci. 17, 637–642. 10.1080/00071667608416320 1000328

[B16] Follett B. K. Mattocks P. W. Farner D. S. (1974). Circadian function in the photoperiodic induction of gonadotropin secretion in the white-crowned sparrow, Zonotrichia leucophrys gambelii. Proc. Natl. Acad. Sci. U. S. A. 71, 1666–1669. 10.1073/pnas.71.5.1666 4525285 PMC388298

[B17] Follett B. K. Scanes C. G. Cunningham F. J. (1972). A radioimmunoassay for avian luteinizing hormone. J. Endocrinol. 52, 359–378. 10.1677/joe.0.0520359 5015389

[B18] Fraps R. M. (1954). Neural basis of diurnal periodicity in release of ovulation-inducing hormone in fowl. Proc. Nat. Acad. Sci. 40, 348–356. 10.1073/pnas.40.5.348 16589486 PMC534134

[B19] Fraps R. M. (1965). Twenty-four-Hour periodicity in the mechanism of pituitary gonadotrophin release for follicular maturation and ovulation in the chicken. Endocrinology 77, 5–18. 10.1210/endo-77-1-5

[B20] Furr B. J. A. Bonney B. C. England R. J. Cunningham F. J. (1973). Luteinizing hormone and progesterone in peripheral blood during the ovulatory cycle of the hen, *Gallus domesticus* . J. Endocrinol. 57, 159–169. 10.1677/joe.0.0570159 4701166

[B21] Gow C. B. Sharp P. J. Carter N. B. Scaramuzzi R. J. Sheldon B. L. Yoo B. H. (1985). Effects of selection for reduced oviposition interval on plasma concentrations of luteinising hormone during the ovulatory cycle in hens on a 24 h lighting cycle. Br. Poult. Sci. 26, 441–451. 10.1080/00071668508416834 4075186

[B22] Hao E.-y. Chen H. Wang D.-H. Huang C.-x. Tong Y.-g. Chen Y.-f. (2020). Melatonin regulates the ovarian function and enhances follicle growth in aging laying hens via activating the mammalian target of rapamycin pathway. Poult. Sci. 99, 2185–2195. 10.1016/j.psj.2019.11.040 32241504 PMC7587849

[B23] Haugen M. J. Johnson A. L. (2010). Bone morphogenetic protein 2 inhibits FSH responsiveness in hen granulosa cells. Reproduction 140, 551–558. 10.1530/REP-10-0211 20639315

[B24] Johnson P. A. van Tienhoven A. vAn Tienhoven A. (1984). Investigations of the significance of the crepuscular LH peak in the ovulatory cycle of the hen (*Gallus domesticus*). J. Endocrinol. 100, 307–313. 10.1677/joe.0.1000307 6538218

[B25] Kim D. Johnson A. L. (2018). Differentiation of the granulosa layer from hen prehierarchal follicles associated with follicle-stimulating hormone receptor signaling. Mol. Reprod. Dev. 85, 729–737. 10.1002/mrd.23042 29995345

[B26] Kim D. Johnson A. L. (2016). Vasoactive intestinal peptide promotes differentiation and clock gene expression in granulosa cells from prehierarchal follicles. Mol. Reprod. Dev. 83, 455–463. 10.1002/mrd.22641 27021352

[B27] Kim D. Lee J. Johnson A. L. (2016). Vascular endothelial growth factor and angiopoietins during hen ovarian follicle development. Gen. Comp. Endocrinol. 232, 25–31. 10.1016/j.ygcen.2015.11.017 26996428

[B28] Kim D. Ocón-Grove O. Johnson A. L. (2013). Bone morphogenetic protein 4 supports the initial differentiation of hen (*Gallus gallus*) granulosa cells. Biol. Reprod. 88, 161. 10.1095/biolreprod.113.109694 23658430

[B29] Knight P. G. Wilson S. C. Gladwell R. Y. Cunningham F. J. (1982). Evidence for the involvement of central catecholaminergic mechanisms in mediating the preovulatory surge of luteinizing hormone in the domestic hen. J. Endocrinol. 94, 295–304. 10.1677/joe.0.0940295 6125554

[B30] Li D. Y. Wu N. Tu J. B. Hu Y. D. Yang M. Y. Yin H. D. (2015). Expression patterns of melatonin receptors in chicken ovarian follicles affected by monochromatic light. Genet. Mol. Res. 14, 10072–10080. 10.4238/2015.August.21.14 26345944

[B31] Li L. Zhang Z. Peng J. Wang Y. Zhu Q. (2014). Cooperation of luteinizing hormone signaling pathways in preovulatory avian follicles regulates circadian clock expression in granulosa cell. Mol. Cell. Biochem. 394, 31–41. 10.1007/s11010-014-2078-3 24825178

[B32] Liu H.-K. Long D. W. Bacon W. L. (2001b). Concentration change patterns of luteinizing hormone and progesterone and distribution of hierarchical follicles in normal and arrested laying Turkey hens. Poult. Sci. 80, 1509–1518. 10.1093/ps/80.10.1509 11599712

[B33] Liu H.-K. Long D. W. Bacon W. L. (2002). Interval between preovulatory surges of luteinizing hormone increases late in the reproductive period in Turkey hens. Biol. Reprod. 66, 1068–1075. 10.1095/biolreprod66.4.1068 11906927

[B34] Liu H.-K. Long D. W. Bacon W. L. (2001a). Preovulatory luteinizing hormone surge interval in old and young laying Turkey hens early in the egg production period. Poult. Sci. 80, 1364–1370. 10.1093/ps/80.9.1364 11558924

[B57] Maddineni S. Metzger S. Ocón O. Hendricks G. Ramachandran R. (2005). Adiponectin gene is expressed in multiple tissues in the chicken: food deprivation influences adiponectin messenger ribonucleic acid. Endocrinology 146, 4250–4256. 10.1210/en.2005-0254 15976057

[B35] McGuire N. L. Kangas K. Bentley G. E. (2011). Effects of melatonin on peripheral reproductive function: Regulation of testicular GnIH and testosterone. Endocrinology 152, 3461–3470. 10.1210/en.2011-1053 21771888

[B36] Melek O. Morris T. R. Jennings R. C. (1973). The time factor in egg formation for hens exposed to ahemeral light‐dark cycles. Br. Poult. Sci. 14, 493–498. 10.1080/00071667308416056 4748870

[B37] Menet J. S. Pescatore S. Rosbash M. (2014). CLOCK:BMAL1 is a pioneer-like transcription factor. Genes Dev. 28, 8–13. 10.1101/gad.228536.113 24395244 PMC3894415

[B38] Morris T. R. (1973). The effects of ahemeral light and dark cycles on egg production in the fowl. Poult. Sci. 52, 423–445. 10.3382/ps.0520423 4575487

[B39] Ocón-Grove O. M. Poole D. H. Johnson A. L. (2012). Bone morphogenetic protein 6 promotes FSH receptor and anti-Müllerian hormone mRNA expression in granulosa cells from hen prehierarchal follicles. Reproduction 143, 825–833. 10.1530/REP-11-0271 22495888

[B40] Rothchild I. Fraps R. M. (1949). The induction of ovulating hormone release from the pituitary of the domestic hen by means of progesterone. Endocrinology 44, 141–149. 10.1210/endo-44-2-141 18109107

[B41] Rozenboim I. Silsby J. L. Tabibzadeh C. Pitts G. R. Youngren O. M. El Halawani M. E. (1993). Hypothalamic and posterior pituitary content of vasoactive intestinal peptide and gonadotropin-releasing hormones I and II in the Turkey hen. Biol. Reprod. 49, 622–626. 10.1095/biolreprod49.3.622 8399858

[B42] Rzasa J. Williams J. Etches R. J. (1983). A study of the ovulation-inhibiting effects of dexamethasone in the domestic hen. Gen. Comp. Endocrinol. 52, 311–314. 10.1016/0016-6480(83)90126-0 6654039

[B43] Sechman A. Paczoska-Eliasiewicz H. Rzasa J. Hrabia A. (2000). Simultaneous determination of plasma ovarian and thyroid hormones during sexual maturation of the hen (*Gallus domesticus*). Folia Biol. 48, 7–12. 11080911

[B44] Sechman A. Pawlowska K. Rzasa J. (2009). Influence of triiodothyronine (T(3)) on secretion of steroids and thyroid hormone receptor expression in chicken ovarian follicles. Domest. Anim. Endocrinol. 37, 61–73. 10.1016/j.domaniend.2009.03.001 19394185

[B45] Sechman A. (2013). The role of thyroid hormones in regulation of chicken ovarian steroidogenesis. Gen. Comp. Endocrinol. 90, 68–75. Epub 2013 Apr 28. 10.1016/j.ygcen.2013.04.012 23631902

[B46] Soliman K. F. A. Huston T. M. (1974). Involvement of the adrenal gland in ovulation of the fowl. Poult. Sci. 53, 1664–1667. 10.3382/ps.0531664 4371216

[B47] Sun S. El Halawani M. E. (1995). Protein kinase-C mediates chicken vasoactive intestinal peptide stimulated prolactin secretion and gene expression in Turkey primary pituitary cells. Gen. Comp. Endocrinol. 99, 289–297. 10.1006/gcen.1995.1112 8536940

[B48] Thayananuphat A. Youngren O. M. Kang S. W. Bakken T. Kosonsiriluk S. Chaiseha Y. (2011). Dopamine and mesotocin neurotransmission during the transition from incubation to brooding in the Turkey. Horm. Behav. 60, 327–335. 10.1016/j.yhbeh.2011.06.009 21741977

[B49] Tischkau S. A. Howell R. E. Hickok J. R. Krager S. L. Bahr J. M. (2011). The luteinizing hormone surge regulates circadian clock gene expression in the chicken ovary. Chronobiol. Int. 28, 10–20. 10.3109/07420528.2010.530363 21182400 PMC9551702

[B50] Warren D. C. Scott H. M. (1935). The time factor in egg formation. Poult. Sci. 14, 195–207. 10.3382/ps.0140195

[B51] Williams J. B. Etches R. J. Rzasa J. (1985). Induction of a pause in laying by corticosterone infusion or dietary alterations: Effects on the reproductive system, food consumption and body weight. Br. Poult. Sci. 26, 25–34. 10.1080/00071668508416783 3971194

[B52] Wilson S. C. Cunningham F. J. Morris T. R. (1982). Diurnal changes in the plasma concentrations of corticosterone, luteinizing hormone and progesterone during sexual develop- ment and the ovulatory cycle of Khaki Campbell ducks. J. Endocrinol. 93, 267–277. 10.1677/joe.0.0930267 7201002

[B53] Wilson S. C. Jennings R. C. Cunningham F. J. (1985). Effects of an advance of darkness on the ovulatory cycle of the hen. Br. Poult. Sci. 26, 83–96. 10.1080/00071668508416790 3971196

[B54] Wilson S. C. Sharp P. J. (1975). Changes in plasma concentrations of luteinizing hormone after injection of progesterone at various times during the ovulatory cycle of the domestic hen (*Gallus domesticus*). J. Endocrinol. 67, 59–70. PMID: 1194828. 10.1677/joe.0.0670059 1194828

[B55] Wilson S. C. Sharp P. J. (1976). Induction of luteinizing hormone release by gonadal steroids in the ovariectomized domestic hen. J. Endocrinol. 71, 87–98. 10.1677/joe.0.0710087 978121

[B56] Zhang L. Chen F. Cao J. Dong Y. Wang Z. Hu M. (2017). Green light inhibits GnRH-I expression by stimulating the melatonin-GnIH pathway in the chick brain. J. Neuroendocrinol. 29. 10.1111/jne.12468 28295740

